# Quality of Life after Venous Stenting for Post-thrombotic Syndrome and the Effect of Inflow Disease

**DOI:** 10.1177/15385744231225802

**Published:** 2023-12-29

**Authors:** Jay M. Bakas, Catherine van Montfrans, Adriaan Moelker, Renate R. van den Bos, Wendy S. J. Malskat, Hence J. M. Verhagen, Marie Josee E. van Rijn

**Affiliations:** 1 Department of Vascular and Endovascular Surgery, 273243 Erasmus Medical Center, Rotterdam, The Netherlands; 2 Department of Dermatology, 6993 Erasmus Medical Center, Rotterdam, The Netherlands; 3 Department of Radiology and Nuclear Medicine, 6993 Erasmus Medical Center, Rotterdam, The Netherlands

**Keywords:** patient reported outcomes, long-term outcomes, quality of life, postthrombotic syndrome, stents

## Abstract

**Objective:**

Patients with PTS experience an impaired quality of life (QoL). We aimed to study QoL in patients stented for post thrombotic syndrome (PTS) and analyze the influence of different parameters.

**Methods:**

Patients stented for PTS after iliofemoral deep vein thrombosis were asked to complete the Chronic Venous Disease Quality of Life Questionnaire (CIVIQ-20) and the Short Form Health Survey (SF-36) in this cross-sectional study. All other data were collected retrospectively. Primary endpoints were median CIVIQ-20 and physical (PCS) and mental (MCS) component summary SF-36 scores. The influence of age, sex, and years between the procedure and completion of questionnaire were investigated using a multivariate linear regression model. Wilcoxon signed rank test compared the PCS and MCS with the normative. Effects of inflow from the deep femoral vein (DFV) and/or the femoral vein (FV) on QoL was analyzed in patients with patent stents.

**Results:**

The response rate was 70.3% (n = 45/64). Time period (median) from stenting to questionnaire completion was 6.6 years (IQR: 8.0). Most stents were placed unilateral left-sided (73.3%). For patients with patent stents (n = 42) median CIVIQ-20 was 35.5 (IQR: 17.3), higher than the minimum of 20.0 (*P* < .001). Median PCS of 44.7 (IQR: 14.2) was lower (*P* < .001), and MCS of 55.9 (IQR: 7.1) higher (*P* = .001) than the normative (50.0). Time since stenting and sex were not associated with QoL. Age was a significant predictor [standardized coefficient ß = .36, *P* = .04] for QoL using the CIVIQ-20, but not for the SF-36. Inflow disease did not impact QoL, but patients with occluded stents (n = 3) had poor functioning levels.

**Conclusion:**

Quality of life is impaired after venous stenting for PTS, particularly physical functioning, among patients with an open stent, but was similar between patients with good and impaired inflow. Patients with a permanent stent occlusion had the lowest QoL.

## Introduction

Post-thrombotic syndrome (PTS) is the most frequent chronic complication of acute deep vein thrombosis (DVT), occurring in 20 to 50% of iliofemoral DVT patients.^1^ Symptoms and signs usually arise within the first year, but may also start decades after the initial DVT.^2^ Preventing DVT and complications, such as PTS, is important based on clinical and economic grounds,^3^ especially considering the disease’s incurability and great impact on relatively young patients.

Patients with PTS experience an impaired quality of life (QoL), which can be similar to other chronic diseases, such as diabetes mellitus, obstructive lung disease, and congestive heart failure.^4^ Treatment mainly focusses on the relief of complaints and prevention of a new DVT by optimized anticoagulation, compression therapy, and lifestyle changes. According to current guidelines of the European Society for Vascular Surgery (ESVS), endovenous treatment should additionally be considered in symptomatic patients with iliofemoral outflow obstruction.^5^

There are various predictors for a poor QoL after DVT, such as development and severity of PTS, iliofemoral DVT, inadequate anticoagulant treatment and incompliance to compression therapy.^6^ Residual thrombus seems related to impaired QoL^7^ and the risk of PTS development is higher in patients with a more proximal DVT compared to patients with a distal DVT.^8^ Patient-reported outcome measures (PROMs) successfully improved after venous stenting between 3 and 24 months of follow-up in patients with PTS^9,10^ but the long-term effects are unknown. Whether venous stenting is beneficial for patients with inflow disease, in terms of QoL, is also unknown.

This cross-sectional study evaluates QoL in PTS patients after stenting for iliofemoral outflow obstruction. All other data were collected retrospectively. Years between the procedure and QoL evaluation, as well as inflow from the deep femoral vein (DFV) and/or femoral vein (FV), were studied to evaluate the long-term effect of venous stenting on QoL in patients with patent stents.

## Methods

### Cohort

All patients with deep venous stents for PTS in the iliac, iliofemoral and/or caval veins were retrospectively screened from the hospital’s first procedure in May 2006 until November 2021. Exclusion criteria were inadequate understanding of the Dutch language, cognitive impairment, or impossible to invite (eg, death or living abroad). The remaining patients were asked for informed consent to complete two questionnaires: the Chronic Venous Disease Quality of Life Questionnaire (CIVIQ-20) and the Short Form Health Survey (SF-36). Patients with informed consent and completed questionnaires were included in this cross-sectional study. The questionnaires were filled out once at least 3 months after the primary procedure in a cross-sectional design, with varying time-intervals for each patient. Non-responders and patients with incomplete questionnaires were re-contacted once, and excluded if no response was sent back within at least 3 months. This study was approved by the local Medical Ethical board (MEC-2020-0429).

Baseline characteristics were retrospectively collected from medical records: age, sex, body mass index (BMI) during the primary procedure, medical history of diabetes mellitus (DM), medical history of peripheral arterial occlusive disease (PAOD), stent laterality, post-stent anticoagulant therapy, and smoking. All patients underwent recent evaluation of venous stent patency, since it was prospectively recorded as a component of a larger study in which the current study was integrated.^11^ The STROBE statement for observational studies was used to report the data.^12^

### Definitions

Open stents were defined as either primary (open without re-intervention), or secondary patent (open after re-intervention). To assess this, all patients underwent duplex ultrasound (DUS). A small proportion underwent additional contrasted computed tomography venography in case of inconclusive DUS. The term permanent occlusion was used for occluded stents without further options to restore patency. Age was reported in years at time that the questionnaires were completed. Inflow was scored by three authors (JB, AM, and MR) using pre- and/or peri-procedural imaging, including duplex ultrasound, magnetic resonance imaging, computed tomography, phlebography, and/or intravascular ultrasound findings. Inflow disease was defined as impeded flow (presence of post-thrombotic changes) from the DFV and/or FV of the affected limb(s) and discrepancies were resolved after reaching 100% negotiated consensus. Patients with good inflow (absence of post-thrombotic changes) from the DFV and FV of the affected limb(s) were categorized as having no inflow disease.

### Stent Procedure

Patients with moderate to severe PTS (Villalta ≥10), or severe venous claudication, were stented at least 1 year after the initial DVT if iliofemoral and/or caval obstruction was present. All patients received low-molecular-weight heparin after venous stenting and switched to a vitamin-K-antagonist or direct-oral-anticoagulant after 2 weeks.

### Quality of Life

Chronic Venous Disease Quality of Life Questionnaire (CIVIQ-20) is a validated 20-question disease-specific questionnaire, which assesses lower limb complaints, impact on daily activities, and mental state in chronic venous disease.^13^ It depicts 4 subdomains (leg-pain, physical activity, psychological activity, and social activity) and a total score. The total score ranges between 20 (no complaints) and 100, with *higher* scores indicating more complaints and a *worse* QoL.

The SF-36 is a validated 36-question survey for health-related QoL. It yields 8 domains (physical functioning, role-physical, bodily pain, general health, vitality, social functioning, role-emotional, and mental health), and a mental and physical component summary (MCS and PCS).^14^ Each domain scores on a scale of 0 to 100, with *higher* scores indicating a *better* QoL, as opposed to the CIVIQ-20. The component summaries are normative-based scores. A component summary of 50 is used as a reference norm for the general population of the United States (US).

### Statistical Analysis

Data were analyzed using SPSS software version 28.0.1.0 (IBM®). The questionnaire responses and demographics were analyzed using descriptive frequencies. Continuous variables were presented as mean with standard deviation (SD), or median with interquartile range (IQR). Scatterplots and boxplots were used to further visualize patient-reported outcomes. Multivariate regression model was used to test if age, sex, and time between venous stenting and questionnaire completion significantly predicted PROMs, using ANOVA-test to calculate *P*-values. The SF-36 component summaries were compared with the normative value of 50.0 for the general population of the United States, using a Wilcoxon signed rank test. The SF-36 and CIVIQ-20 outcomes were also compared between patients with and without inflow disease after successful venous stenting (patent stents). The Shapiro-Wilks test was used to test if samples were normally distributed. Non-parametric independent *t*-test was used to calculate *P*-values, with statistical significance set at *P* < .05. Missing data were excluded from the analysis.

## Results

### Cohort

**Figure 1.**
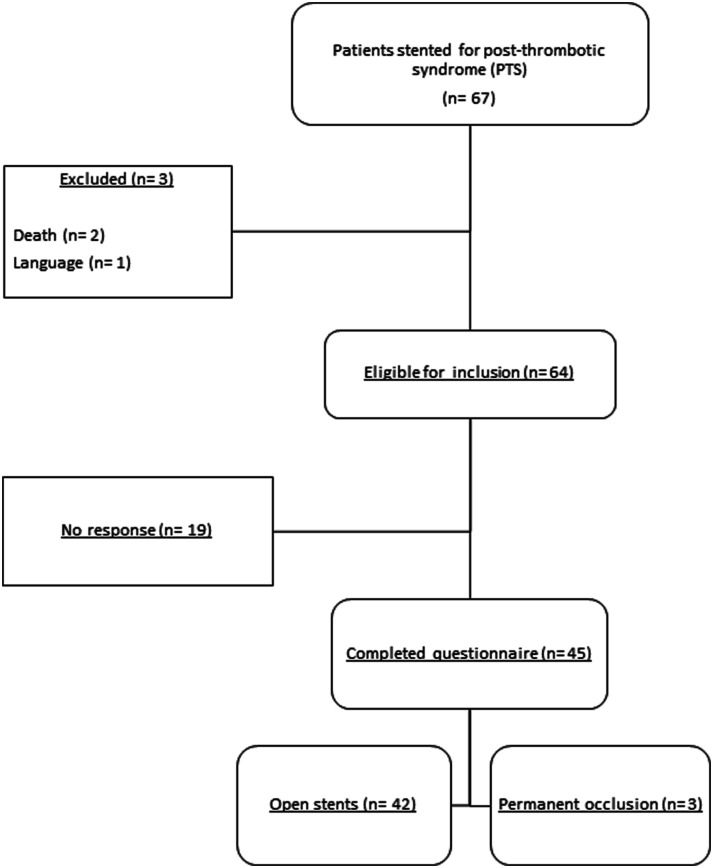
Flowchart patient inclusion.

A total of 67 patients underwent venous stenting for PTS, as shown in Figure 1. Three patients were excluded and the remaining 64 patients were invited to complete the CIVIQ-20 and SF-36 questionnaires, which resulted in a response rate of 70.3% (n = 45/64). Characteristics of included patients are shown in Table 1. Mean age at time of questionnaire completion was 54 years, most patients were female (80%), and most stents were placed unilateral left-sided (73.3%). None of the patients had medical history of PAOD, and one patient had diabetes mellitus. Time period (median) from venous stenting to questionnaire completion was 6.6 years (IQR: 8.0). Permanent stent occlusion was observed in 3 patients. Data were missing for BMI (n = 8 patients) and smoking (n = 3 patients).

**Table 1.** Characteristics of Included Patients.

	Post-thrombotic syndrome (n = 45)
Age, years	54 ± (SD 14)
Female	36 (80.0)
BMI *	25.7 (IQR 5.4)
Diabetes	1 (2.2)
PAOD	0 (0)
Smoking *	17 (40.5)
Permanent occlusion	3 (6.7)
Follow-up, years	6.6 (IQR: 8.0)
Stent laterality	
Left (unilateral)	33 (73.3)
Right (unilateral)	3 (6.7)
Bilateral	9 (20.0)
Anticoagulant therapy (post-stent)	
LMWH switched to VKA	41 (91.1)
LMWH switched to DOAC	4 (8.9)

Categorical variables presented as count (%) and continuous variables as mean ± standard deviation (SD) or median with interquartile range (IQR); age and follow-up at time the questionnaire was filled out; PAOD: peripheral arterial occlusive disease; LMWH: low-molecular-weight-heparin; VKA: vitamin-K-antagonist; DOAC: direct-oral anticoagulant; *missing values n = 8 for Body Mass Index (BMI) and n = 3 for smoking.

### Longitudinal Patient-Reported Outcome Measures

**Figure 2.**
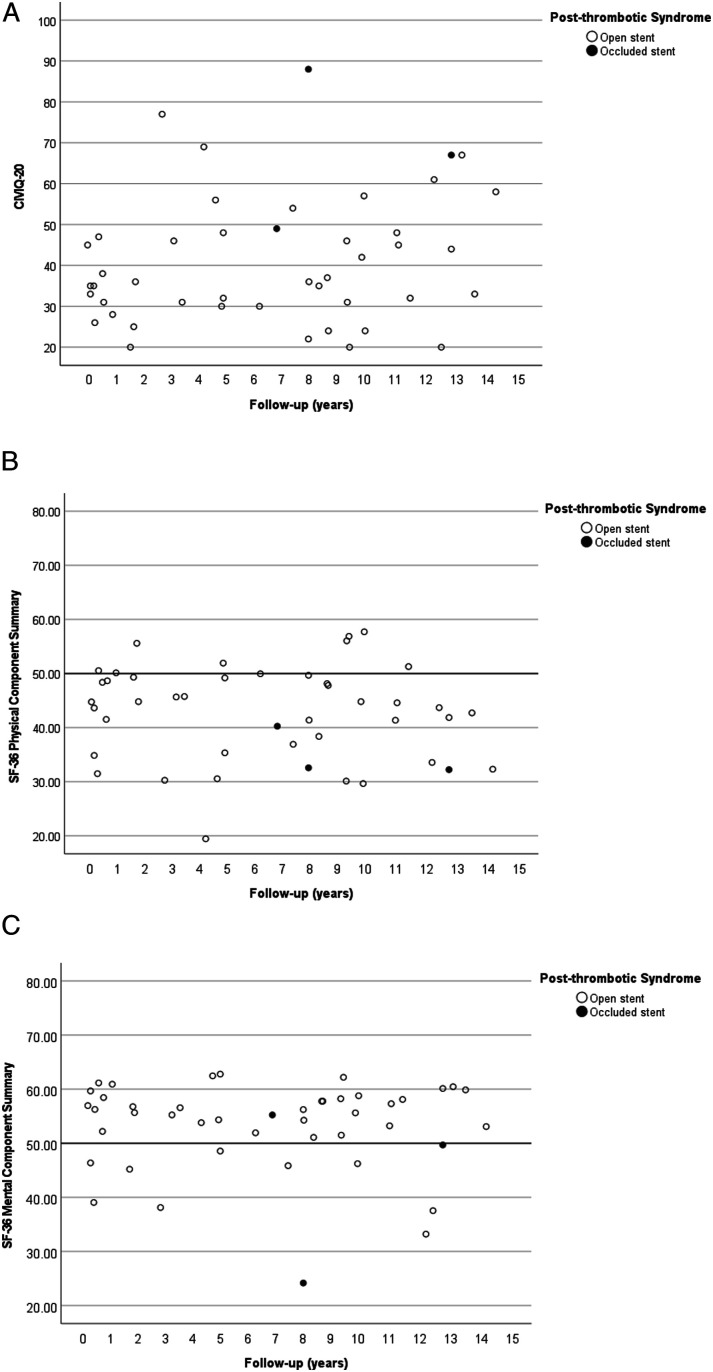
Patient-reported outcome measures in patients with open and occluded venous stents. (A): Total CIVIQ-20 scores plotted against years between the primary procedure and completion of the questionnaire in years. (B): Total SF-36 physical component summary plotted against years between the primary procedure and completion of the questionnaire in years. (C): Total SF-36 mental component summary plotted against years between the primary procedure and completion of the questionnaire in years.

Figure 2(A) displays a scatterplot for total CIVIQ-20 score (*y*-axis) vs years between the primary procedure and completion of the questionnaire (*x*-axis) for patients with open and occluded stents. It shows wide ranges of CIVIQ-20 scores between PTS patients with patent stents. The median CIVIQ-20 of patients with patent stents (n = 42) was 35.5 (IQR: 17.3), significantly higher than the minimum of 20.0 (*P* < .001). The patients with permanent stent occlusion were in the subgroup of patients with the highest CIVIQ-20 scores, indicating more complaints and disabilities of chronic venous disease than patients with open stents (see paragraph 3.3). Sex [standardized coefficient ß = .007, *P* = .96] and years between venous stenting and questionnaire completion [standardized coefficient ß = .30, *P* = .08] did not significantly predict higher CIVIQ-20 (not shown). Age was a significant predictor for CIVIQ-20 [standardized coefficient ß = .36, *P* = .04], indicating higher (thus worse) CIVIQ-20 in older patient.

Figure 2(B) shows a scatterplot for SF-36 PCS for each included patient. Median PCS of patients with patent venous stents (n = 42) was 44.7 (IQR: 14.2), significantly lower than the normative value of 50.0 (*P* < .001). Patients with permanent stent occlusion reported the lowest SF-36 scores, indicating more physical impairments than patients with open stents (see paragraph 3.3). Age [standardized coefficient ß = −.28, *P* = .10], sex [standardized coefficient ß = −.22, *P* = .17], and years between venous stenting and questionnaire completion [standardized coefficient ß = −.24, *P* = .15] did not significantly predict SF-36 PCS (not shown).

Figure 2(C) depicts a similar scatterplot for the SF-36 MCS. The median score of MCS was 55.9 (IQR: 7.1) significantly higher than the normative value of 50.0 (*P* = .001). The MCS was scattered for patients with permanent occlusion, with one scoring above, one around, and one below the reference of 50.0. Age [standardized coefficient ß = −.03, *P* = .86], sex [standardized coefficient ß = −.12, *P* = .47], and years between venous stenting and questionnaire completion [standardized coefficient ß = −.01, *P* = .96]. Did not significantly predict SF-36 MCS (not shown).

### Inflow Disease

Out of all 42 patients with patent venous stents, 24 had impaired inflow from the DFV and/or FV. The remaining 18 patients had good inflow from both veins. Outcomes of the (subdomains of) SF-36 and CIVIQ-20, between patients with and without inflow disease, are compared in Table 2. For patients without inflow disease, the median PCS was 44.1 (IQR: 9.5), median MCS 56.1 (IQR: 8.8), and total CIVIQ-20 was 34.0 (IQR: 22.0), after a median time of 8.8 years after venous stenting (IQR: 9.5). No significant differences were found compared to patients with inflow disease (median PCS of 43.8 (IQR: 16.8), median MCS of 55.9 (IQR: 7.1), and total CIVIQ-20 was 36.0 (IQR: 22.3)), after a median time of 5.4 years after venous stenting (IQR: 7.6). Also no differences were found between any of the SF-36 subdomains.

**Table 2.** Patient Reported Outcomes for Patients With Patent Venous Stents: With and Without Inflow Disease of the (Deep) Femoral Vein.

	Good inflow DFV/FV (n = 18)	Inflow disease DFV/FV (n = 24)	*P*-value
**SF-36**			
**Physical functioning** (0-100)	77.5 (IQR: 36.3)	75.0 (IQR: 45.0)	.15
**Role-physical** (0-100)	100 (IQR: 56.3)	100 (IQR: 50.0)	.77
**Bodily pain** (0-100)	62.0 (IQR: 14.8)	72.0 (IQR: 30.3)	.58
**General health** (0-100)	58.5 (IQR: 29.5)	59.5 (IQR: 33.3)	.92
**Vitality** (0-100)	55.0 (IQR: 17.5)	67.5 (IQR: 33.3)	.31
**Social functioning** (0-100)	81.3 (IQR: 38.1)	87.5 (IQR: 46.9)	.49
**Role-emotional** (0-100)	100 (IQR: 8.3)	100 (IQR: 25.0)	.84
**Mental health** (0-100)	82.0 (IQR: 17.0)	84.0 (IQR: 12.0)	.76
**Physical component summary** (0-100; 50.0 = US reference norm)	44.1 (IQR: 9.5)	43.8 (IQR: 16.8)	.88
**Mental component summary** (0-100; 50.0 = US reference norm)	56.1 (IQR: 8.8)	55.9 (IQR: 7.1)	.99
**CIVIQ-20**			
**Leg pain** (Q1-4)	8.0 (IQR: 5.5)	8.0 (IQR: 4.8)	.53
**Physical activity** (Q5-7, 9)	8.0 (IQR: 5.5)	7.0 (IQR: 6.0)	.91
**Psychological activity** (Q12-20)	15.5 (IQR: 7.5)	16.0 (IQR: 8.5)	.21
**Social activity** (Q8, 10, 11)	5.0 (IQR: 5.3)	5.5 (IQR: 5.0)	.26
**Total score** (20-100)	34.0 (IQR: 22.0)	36.0 (IQR: 22.3)	.45
Follow-up * Years	8.8 (IQR: 9.5)	5.4 (IQR: 7.6)	.66

Variables presented as median with interquartile range (IQR). DFV = deep femoral vein. FV = femoral vein. Q = question. US = United States. * between stenting procedure and questionnaire completion.

### Permanent Stent Occlusion

Table 3 shows patients with a permanent stent occlusion (n = 3). Two were female (32 and 63 years old) and one male (49 years old). Time between the primary stent procedure and completion of the questionnaire were 6.7, 12.7, and 7.8 years. The reported CIVIQ-20 scores were 49, 67, and 88, which are all three above the median CIVIQ-20 for patients with patent venous stents (35.5, IQR: 17.3). The physical component summaries (40.3, 32.2, and 32.6) were below the reference normative of 50, and also below the median of patients with patent venous stents (44.7, IQR: 14.2). The median MCS was 24.2 for one patient with multiple comorbidities and similar or above the reference norm of 50.0 for the other two patients (scores of 55.2 and 49.7). All three MCS of patients with permanent stent occlusion were below the median score of patients with patent venous stents 55.9 (IQR: 7.1). Permanent stent occlusion was likely related to pre-existent poor inflow for two and inability to restart anticoagulant therapy for one patient.

**Table 3.** Characteristics and Outcomes of Patients With a Permanent Stent Occlusion.

Patient	Remarks	Patient reported outcome measures
**Female patient**	**Stent**	**CIVIQ-20**
32 years old	• Wallstent	total score: 49
PTS, left limb	• Placed across the inguinal ligament	
	• Good inflow DFV and FV.	
	**Hematological**	**SF-36**
	• Heterozygous MTHFR mutation	mental component summary: 55.2
	• Anticoagulants: VKA	Physical component summary: 40.3
	**Comorbidity**: Multiple VTE, complicated pregnancy, miscarriage.	**Follow-up**
	**Cause:** Cessation of anticoagulant therapy because of blood loss during pregnancy	6.7 years
**Female patient**	**Stent**	**CIVIQ-20**
63 years old	• Wallstent, smart and SinusXL stents.	total score: 67
PTS, bilateral	• Both sides placed above the inguinal ligament	
	• Impaired inflow of left DFV and FV.	
	Good inflow of the right DFV and FV.	
	**Hematological**	**SF-36**
	• Antiphospholipid syndrome	mental component summary: 49.7
	• Anticoagulants: VKA	Physical component summary: 32.2
	**Comorbidity:** Multiple VTE, alopecia androgenica, hirsutism, monoclonal gammopathy.	**Follow-up**
	**Cause:** Pre-existent poor stent inflow	12.7 years
**Male patient**	**Stent**	**CIVIQ-20**
49 years old	• Wallstent	total score: 88
PTS, left limb	• Placed across the inguinal ligament	
	• Poor inflow of the DFV, impaired inflow of the FV	
	**Hematological**	**SF-36**
	• Thrombophilia not tested.	mental component summary: 24.2
	• Anticoagulants: Dual antiplatelet and VKA.	Physical component summary: 32.6
	**Comorbidity:** Former addiction to intravenous drugs, myocardial infarct, endocarditis, osteomyelitis, thoracic empyema, femoral fractures treated by surgical reposition and fixation.	**Follow-up**7.8 years
	**Cause:** Pre-existent poor stent inflow	

Age and follow-up at time the questionnaire was filled out. PTS: post-thrombotic syndrome. VTE: venous thromboembolism. VKA: vitamin-K-antagonist; DFV = deep femoral vein; FV: femoral vein.

## Discussion

Impaired QoL was found in PTS patients particularly in terms of physical functioning, regardless of long-term stent patency. Mental disabilities were less common after venous stenting and QoL was not influenced by the time interval between the procedure and questionnaire completion. Patients with inflow disease, but open stents, had a similar QoL as patients with a good inflow. Patients with a permanently occluded stent had the poorest QoL.

Median PCS was below the reference value of 50 in the first three quartiles (75.0%) of our cohort. Improved scores were previously reported on several SF-36 domains after venous stenting for PTS, such as physical functioning, role-physical, and bodily pain.^15^ These results kept improving after two years of follow-up after venous stenting.^10^ In our study, we did not find a relationship between the time of questionnaire completion and SF-36 component summaries. This suggests that patients reach a stable phase with consistent functioning levels, where the timing of assessment after stent placement no longer impacts QoL. However, this observation should be considered an assumption, because the different time intervals were not assessed in the same patients, but across patients.

Improved outcomes after stenting were also reported in two studies using the CIVIQ-20 questionnaire, although the follow-up was limited to 3 and 12 months.^16,17^ Patients with patent stents from the present study had comparable CIVIQ-20 to those reported in previous studies after venous stenting. The time of questionnaire completion again not significantly related to QoL when using the CIVIQ-20 questionnaire in our cohort.

The physical impairments in a considerable group of PTS patients did not seem to be attributable to inflow disease. There were no significant differences in PROMs between patients with good and impaired inflow, in case of patents stents. This may be explained by the higher risk of PTS development in patients with a more proximal DVT compared to patients with a distal DVT.^8^ From a hemodynamical perspective, the relief of iliofemoral outflow obstruction in patients with inflow disease, is likely to change them into ‘distal DVT’ patients if their stents remain open, also in terms of QoL.

While future studies are needed to identify patients who will benefit the most from venous stents (eg, by predicting QoL), this study reveals that physical disabilities are common in endovascular treated PTS patients, at different time points after the procedure, even if stents are patent. These results emphasize the importance of continuously monitoring QoL during follow-up after venous stenting. Adjunctive treatment measures, such as compression therapy and lifestyle changes, should be specifically encouraged in patients with lowered QoL due to physical disabilities. Patients with PTS who were compliant to compression stockings had similar QoL scores to patients without PTS in a previous study.^18^ Additionally, physical activity might be promoted by the use of smart watches and similar devices, which are useful for both monitoring and supporting healthy lifestyle interventions.^19^

The poorest QoL was found in patients with a permanent stent occlusion. Previously, reasons for re-thrombosis were classified and scored as related to technical issues, poor inflow, coagulation disorders, and/or inadequate anticoagulant therapy. Reasons why re-opening was not possible in these 3 patients were poor inflow for two and inability to restart anticoagulant therapy for one patient. Our findings emphasize the importance of preserving stent patency specifically by paying attention to inflow and optimal anticoagulant treatment. In cases where the loss of patency is likely, invasive treatment should be avoided.

This study has several limitations. The cross-sectional study design prevented us from comparing QoL before and after venous stenting. The included cohort spans over 15 years of time, presumably leading to heterogeneity in patient selection, treatment technique, and follow-up. The long time interval between the procedure and questionnaire completion is at the same time the unique part of the study. The small size of the groups limited the ability to adjust for potential confounders, although the poorest QoL was found after permanent occlusion, suggesting to support an association between venous patency and PTS severity.^20^ While inflow was measured before and during the procedure, allocation to presence or absence of inflow disease was performed retrospectively for the purpose of this study. It is unknown if QoL differs between patients who denied study participation and those who consented, but the response rate suggests a low likelihood of non-response bias, which seems good compared to other studies.^21,22^

## Conclusion

Impaired physical functioning is common after endovenous stenting for PTS, independent from time since procedure. Continuous monitoring and consultation during follow-up are necessary to consider adjunctive treatment, regardless of stent patency and inflow disease. Identifying predictors for improved QoL after endovenous interventions are needed to determine suitable candidates for these procedures. Patients with inflow disease may still benefit from venous stenting in terms of QoL, but selection is key as inflow is related to re-interventions for in-stent thrombosis.^11^ Preventing stent occlusion is crucial since patients with occluded stents had poor QoL. To achieve this, it should be recommended to avoid venous stenting in high-risk patients prone to stent failure. Identifying these high-risk patients should be the subject of future studies.
